# Unpacking the Role of YouTube Influencers in Shaping Healthy Attitudes and Behaviors in Saudi Arabia: A Cross-Sectional Study

**DOI:** 10.7759/cureus.57390

**Published:** 2024-04-01

**Authors:** Mohammed A Aljunaid, Najim Z Alshahrani, Mohamed Terra, Mohamed Baklola

**Affiliations:** 1 Department of Family and Community Medicine, Faculty of Medicine, University of Jeddah, Jeddah, SAU; 2 Public Health, Mansoura University, Mansoura, EGY

**Keywords:** health, fashion, youtube™, youtube health, health attitudes, gender disparities, saudi arabia, health behaviors, social media, youtube influencers

## Abstract

Background

Social media, notably YouTube (Google LLC, Mountain View, California, United States), has transformed global communication and access to information. In Saudi Arabia, with high internet usage, YouTube influencers play a significant role in shaping health attitudes and behaviors. This study investigates the impact of YouTube influencers on health behaviors among Saudi Arabian residents, considering the unique cultural and socio-demographic context.

Methods

We conducted a community-based cross-sectional study in Saudi Arabia. We surveyed a convenience sample of 703 young Saudi participants from September 2022 to March 2023. Data collection utilized an anonymous online questionnaire distributed via popular social media platforms. The questionnaire covered sociodemographic information, health habits, perceptions of influencers, and their impact on health attitudes. Descriptive statistics, including percentages, frequencies, means, and standard deviations, were employed to summarize participant characteristics and health-related variables. Pearson’s chi-square test was utilized to compare response variables among different groups.

Results

Findings show significant gender disparities in health habits, with males more likely to smoke but females less physically active (p<0.01). Influencers were perceived as encouraging healthy habits by 71.7% of participants, yet 55.6% also believed they promoted unhealthy habits. Positive outcomes included dietary improvements (62.6%) and smoking cessation (20.5%). People preferred healthcare influencers the most (66.8%), followed by sports and fashion influencers.

Conclusions

This study highlights YouTube influencers' substantial influence on health behaviors in Saudi Arabia. It suggests the potential for influencers, especially in healthcare, to contribute positively to public health. Viewer discernment is essential due to varying influencer impacts. These findings underscore the complex role of YouTube influencers in shaping health behaviors and suggest avenues for targeted health interventions.

## Introduction

Social media platforms have revolutionized the way individuals interact, communicate, and access information in the digital era [1]. Among these platforms, YouTube (Google LLC, Mountain View, California, United States) has emerged as a dominant force, captivating a vast global audience through its diverse range of video content [1,2]. With its widespread popularity, YouTube has become a powerful tool for sharing knowledge, shaping opinions, and influencing behaviors [2]. YouTube is the most popular dedicated video-sharing application, with more than two and a half billion users, or nearly one-third of the world population [3,4]. YouTube reports that hundreds of millions of hours are spent daily on their platform, resulting in billions of views every day [4]. The development of multifunctional digital components and devices has encouraged users to engage with YouTube via different hardware platforms and interfaces, including television, personal computers, laptops, tablets, and smartphones [2]. YouTube offers a vast array of videos covering various topics, including entertainment, education, lifestyle, and health.

Saudi Arabia has experienced a significant increase in the popularity of social media platforms, largely due to its high internet penetration rates and the tech-savvy nature of its population. As of the beginning of 2023, there were 36.31 million internet users in Saudi Arabia, representing a 99% internet penetration rate. In January 2023, the country boasted 29.10 million social media users, which accounted for 79.3 percent of the total population [5]. This places Saudi Arabia among the leading nations globally in terms of social media usage, with YouTube being one of the most commonly used platforms. Particularly among the younger demographic, Saudi Arabian internet users heavily rely on YouTube for information, entertainment, and cultural expression [6].

Notably, YouTube's influence extends beyond mere entertainment value. The platform has played a substantial role in shaping attitudes and behaviors, particularly in relation to health [7-9]. YouTube influencers, individuals who have amassed substantial followings through their videos, hold considerable sway over their viewers. These influencers have harnessed their popularity to disseminate health-related content, provide advice, share personal experiences, and promote specific health behaviors [7]. The impact of YouTube influencers on health attitudes and behaviors has been observed worldwide. Studies have shown that YouTube plays a significant role in influencing individuals' perceptions and decisions regarding health-related topics such as fitness, nutrition, mental health, and preventive care [10-12]. The captivating nature of video content, combined with the trust and relatability established by influencers, has made YouTube a potent medium for health communication.

However, despite the growing recognition of YouTube's influence on health-related attitudes and behaviors, there is a notable gap in understanding the specific dynamics within the Saudi Arabian context. Cultural nuances, religious influences, and the unique sociodemographic characteristics of the Saudi Arabian population may contribute to distinct patterns of YouTube influencer impact on health-related perceptions and behaviors. Therefore, conducting a comprehensive study that focuses on Saudi Arabian audiences is essential to gain insights into this particular context and address the existing knowledge gap.

This study aims to investigate the influence of YouTube influencers on the formation of healthy attitudes and behaviors among residents of Saudi Arabia. It hypothesizes that socio-demographic characteristics, gender-based disparities in lifestyle and health habits, and the perceived impact of influencers will collectively contribute to elucidating the role of influencers in shaping health attitudes and fostering positive behavioral changes within the Saudi Arabian population.

## Materials and methods

Study design and study period

A descriptive, community-based cross-sectional study with an analytic component was conducted among the general population of Saudi Arabia from September 25, 2022, to March 15, 2023. All Saudi individuals aged over 18 years were eligible for inclusion in this study.

Sample size

The minimum required sample size was determined using the Raosoft sample size calculator (Raosoft, Inc., Seattle, Washington, United States) [13]. Assuming a 50% population proportion with a 5% margin of error and a confidence interval of 95%, a sample size of 385 participants was calculated as the sample size for this study.

Sampling and data collection approach

A convenience sampling method was employed to select participants after the required sample size was determined. Participants were invited to complete a structured questionnaire through a designated platform, specifically a Google Forms link. The questionnaire was distributed through various social media platforms, such as WhatsApp (Meta Platforms, Inc., Menlo Park, California, United States), Facebook (Meta Platforms, Inc., Menlo Park, California, United States), and Twitter (Twitter, Inc., San Francisco, California, United States). Before completing the questionnaire, participants were required to provide informed consent by clicking on a consent statement. The statement included an acknowledgment that they had read and understood the purpose of the study and agreed to participate voluntarily. The participants were informed that their responses would be treated confidentially. Upon completion of the survey, participants submitted their responses by clicking on the "submit" icon. Respondents were reminded that all items in the questionnaire needed to be answered for their responses to be considered valid.

Study tools

We utilized an anonymous online questionnaire to collect data. The questionnaire was divided into four sections. The initial section gathered sociodemographic information, including location, gender, age, education, employment, and monthly income. The second section comprised seven multiple-choice questions that assessed the respondents' practices. The third section included 15 multiple-choice questions focused on the characteristics of influencers on social media. Lastly, the fourth section consisted of nine multiple-choice questions that examined the respondents' opinions regarding their attitudes toward influencers on social media. The questions were formulated after conducting an extensive literature search on social media-related studies across various databases. To ensure reliability and clarity, the questionnaire underwent a pre-test with 30 participants from the general population and was reviewed by four professors from the public health and sociology departments. The pilot study results were solely used to enhance the clarity of the questions. In the pilot study, internal consistency was examined using Cronbach’s α reliability coefficient, which was calculated to be 0.71, indicating moderate to good reliability.

Study variables

The study employed a diverse range of variables meticulously chosen to evaluate the impact of YouTube influencers on the formation of healthy attitudes and behaviors among Saudi residents. These variables included age, gender, marital status, residency region, educational level, the field of work, monthly family income, body mass index (BMI), exercise frequency, adherence to a healthy diet, smoking status, overall health condition, the primary source of learning healthy habits, preferred presentation of health-related information on social media, influencers followed, their field of focus, health advice and habits promoted, adherence of influencers to health advice, the influence of influencers on unhealthy habits, presentation of healthy habits by influencers, content related to influencers' fields, trust in non-health field influencers' health advice, motivation and monetary purposes of influencers, experience with health advice from social media, negative results experienced, influencers' impact on good health, the accuracy of influencers' image of a healthy lifestyle, and satisfaction with lifestyle changes.

Statistical analysis

Data were analyzed using IBM SPSS Statistics for Windows, Version 25 (released 2017; IBM Corp., Armonk, New York, United States) [14]. To summarize the demographic characteristics of the participants, descriptive statistical measures (i.e., percentage, frequency, mean, and standard deviation) were used. Pearson’s chi-square test was used to compare response variables and explanatory variables. When the p-value is less than 0.05, the result is reported as statistically significant.

## Results

Socio-demographic and health-related characteristics of study participants

Table 1 shows that the study comprised 703 participants with a median age of 29.2 (standard deviation (SD) ±11.3). The majority were Saudi nationals (90.6%) and females (61%). Over half were single (54.5%) and unemployed (54.6%). In terms of regional distribution, 25.3% were from the Western Area and 22.8% from the Eastern Area, with the rest evenly distributed across other regions. The predominant educational level was a bachelor’s degree (61.2%). Monthly family incomes varied, with the most common bracket being 10,000-20,000 Saudi Riyals (44.5%). The BMI indicated that 61.9% were normal or underweight, while 38.1% were overweight or obese. A majority did not smoke (77.4%), 43.8% reported no physical activity per week, and 57.5% claimed to follow a healthy diet.

**Table 1:** Socio-demographic and health-related characteristics of study participants

Variable		Frequency n(%)
Overall		703 (100)
Age in years (Median ±SD)	29.2 (11.3)	
Nationality	Saudi	637 (90.6)
	Non-Saudi	66 (9.4)
Gender	Male	274 (39)
	Female	429 (61)
Marital status	Single	383 (54.5)
	Married	289 (41.1)
	Others (divorced/widow/widower)	31 (4.4)
Residence	Southern Region	137 (19.5)
	Northern Region	91 (12.9)
	Eastern Region	160 (22.8)
	Western Region	178 (25.3)
	Central Region	137 (19.5)
Educational level	Secondary school	53 (7.5)
	Bachelor’s degree	430 (61.2)
	Post-graduate degree	220 (31.3)
Employment status	Employed	319 (45.4)
	Unemployed	384 (54.6)
Monthly family income (Saudi Riyal)	Less than 3,000	101 (14.4)
	3,000- 10,000	146 (20.8)
	10,000-15,000	155 (22)
	15,000- 20,000	158 (22.5)
	More than 20,000	143 (20.3)
Body mass index (BMI)	Normal‎/Underweight	435 (61.9)
	Overweight‎/Obese	268 (38.1)
Current smoker	Yes	159 (22.6)
	No	544 (77.4)
Frequency of physical activity/week	No physical activity	308 (43.8)
	Once	146 (20.7)
	Two to three times	134 (19.1)
	More than three times	115 (16.4)
Following healthy diet	Yes	404 (57.5)
	No	299 (42.5)

Comparison of lifestyle, health habits, and social media usage for health information based on gender

Gender differences were significant in certain health habits, as shown in Table 2. Male participants were more likely to be current smokers (35.8%) compared to females (14.2%), with a p-value of <0.001. Females were less physically active, with 46.9% reporting no physical activity compared to 38.7% of males (p=0.004). Regarding diet, a higher percentage of males (45.6%) followed a healthy diet compared to females (40.6%), although the difference was not statistically significant (p=0.18). In describing their general health status, a higher proportion of males considered their health to be bad (15%) compared to females (8.4%, p=0.01).

**Table 2:** Comparison of lifestyle, and health habits between participants based on gender * Statistically significant p-value less than 0.05

Variable		Female n (Column%)	Male n (Column%)	P-value
Overall		429 (61)	274 (39)	
Current smoker	Yes	61 (14.2)	98 (35.8)	<0.001*
	No	368 (85.8)	176 (64.2)	
Frequency of physical activity/week	No physical activity	201 (46.9)	106 (38.7)	0.004*
	Once	94 (21.9)	52 (19)	
	Two to three times	80 (18.6)	54 (19.7)	
	More than three times	54 (12.6)	62 (22.6)	
Following healthy diet	Yes	174 (40.6)	125 (45.6)	0.18
	No	255 (59.4)	149 (54.4)	
How do you describe your health status in general?	Bad	36 (8.4)	41 (15)	0.01*
	Good	233 (54.3)	145 (52.9)	
	Excellent	160 (37.3)	88 (32.1)	

Perception and impact of influencers on health attitudes and behaviors

The majority of participants (71.7%) believed that some influencers encourage adopting healthy daily habits, and 52.9% thought that influencers mostly adhere to the health advice they provide. Over half (55.6%) felt that some influencers encourage unhealthy habits. A significant portion (52.1%) observed that the promotion of healthy habits was a common topic presented very often by influencers. In terms of expertise, 65.1% felt that some influencers were experts in the fields they talked about. When it came to the promotion of healthy products, 69.7% believed this was done sometimes for monetary purposes. A sizable group (42.7%) had noticed that influencers committed to proper health habits tend to have good health. Overall, 67% were somewhat satisfied with lifestyle changes due to the influence of social media influencers. Table 3 provides more details.

**Table 3:** Participants’ attitude and beliefs towards YouTube influencers

Variable		Frequency	Column %
In your opinion, do the influencers you follow encourage adopting healthy daily habits?	Some of them	504	71.7
	No	38	5.4
	Neutral	105	14.9
	Yes, all of them	56	8
Do influencers adhere to the health advice they provide to the public?	Sometimes	258	36.7
	Never	16	2.3
	Yes, all the time	57	8.1
	Yes, most of the time	372	52.9
In your opinion, do the influencers you follow encourage unhealthy habits?	Some of them	391	55.6
	No	207	29.4
	Neutral	75	10.7
	Yes, all of them	30	4.3
Do you believe that promoting healthy habits is a common topic presented repeatedly by influencers?	Very often.	366	52.1
	Never	18	2.6
	Rarely	216	30.7
	Yes, always	103	14.7
Are the contents presented by influencers you follow related to their specific fields?	I don't know	48	6.8
	No, none of them are experts in the field they talk about	125	17.8
	Yes, some of them	458	65.1
	Yes, all of them	72	10.2
Do you think that the promotion of healthy products by influencers is solely for monetary purposes?	Sometimes	490	69.7
	No	44	6.3
	Yes, all the time	169	24
Have you noticed that influencers who are committed to proper healthy habits tend to have good health?	Some of them	383	54.5
	No	20	2.8
	Yes	300	42.7
Do you agree influencers represent an accurate image of a healthy lifestyle?	Some of them	519	73.8
	No	108	15.4
	Yes	76	10.8
Overall, to what extent are you satisfied with the changes that have occurred in your lifestyle due to the influence of celebrities on YouTube?	Completely satisfied	122	17.4
	Somewhat satisfied	471	67
	Not satisfied	110	15.6

Positive outcomes experienced by our participants

**Figure 1:**
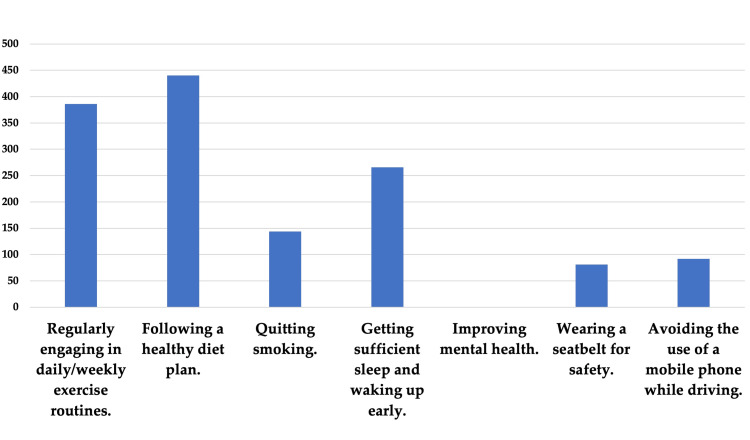
Positive outcomes experienced after adopting health tips from YouTube influencers

Figure 1 illustrates the impact of social media influencers on the participants' health behaviors. The most pronounced positive outcome was the adoption of a healthy diet plan, as reported by 440 participants. A smaller but still notable group of 144 participants successfully quit smoking. Interestingly, none of the participants reported improved mental health as a result of adopting tips from social media influencers.

Distribution of influencer fields based on respondents' preferences

**Figure 2:**
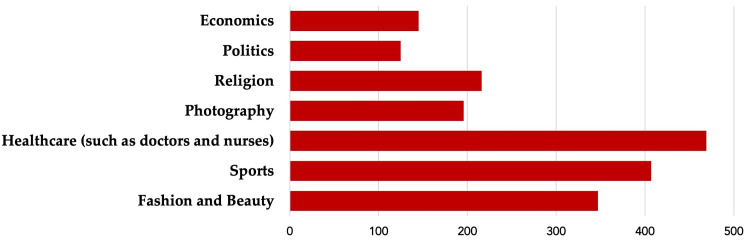
Distribution of YouTube influencer fields or focus based on respondents' preferences

Figure 2 highlights the distribution of fields or areas of focus that respondents prefer among the influencers they follow. Healthcare influencers, including doctors and nurses, were the most preferred, as cited by 469 participants. Sports influencers were the second most popular, with 407 participants indicating a preference for them. Fashion and beauty influencers were also well represented, with 347 participants choosing them. Other fields like photography (196), religion (216), politics (125), and economics (145) had fewer followers among the respondents.

## Discussion

Our study presents a unique analysis of the role of social media influencers, particularly on YouTube, in shaping health attitudes and behaviors among Saudi Arabian residents. The research comprehensively covers socio-demographic characteristics, gender-based health habits, and the overall influence of social media influencers on public health.

The sample predominantly consists of young, educated, and single individuals, which is indicative of the demographic that generally engages more with social media. The representation of 61% females among the participants also falls in line with global observations that women are generally more proactive in seeking health-related information online [15]. The higher prevalence of single and unemployed individuals in the sample suggests that these groups may be more likely to have the time to engage with social media, thereby making them a strategic target for health promotion campaigns [16].

The gender disparity in smoking rates is alarming, with males significantly more likely to smoke compared to females. This finding points to an urgent need for targeted health campaigns. Moreover, the data reveals that females were less physically active than their male counterparts. While societal norms and lifestyle choices might contribute to this pattern, it creates an avenue where influencers can make a positive impact, especially those influencers who are popular among female audiences [15].

Participants appear to hold contrasting views about the role of influencers in encouraging healthy and unhealthy habits. On the one hand, a majority (71.7%) believe that at least some influencers encourage healthy daily habits. On the other hand, 55.6% also feel that some influencers promote unhealthy behaviors. This dual perception may stem from the wide variety of content available on social media platforms [16-18]. The data suggests a need for viewers to have a nuanced understanding and critical evaluation skills when consuming health-related advice online.

Regarding the influence on positive health behaviors, diet, and exercise were the two areas where influencers seemed to have the most positive impact. This finding is likely reflective of the current trend where health and fitness influencers are gaining increasing visibility and following [19,20]. However, it's worth noting that not a single participant reported improvements in mental health. This could indicate a gap in the available content on mental well-being, or perhaps a lack of specialized influencers in the area of mental health.

Interestingly, healthcare influencers were the most popular, followed by those in sports and fashion/beauty. This may be indicative of the general public's trust in influencers who are likely to possess some degree of expert knowledge, especially in health-related fields [21,22]. However, it also highlights an opportunity for influencers in non-traditional sectors such as politics, economics, and religion to contribute positively to public health by incorporating health-promoting messages into their content.

Limitations

Several limitations should be considered when interpreting the findings of this study. First, the cross-sectional nature of the study does not allow for the establishment of causal relationships between exposure to YouTube influencers and changes in health attitudes and behaviors. Secondly, the sample is skewed towards a younger, educated, and primarily single demographic, potentially limiting the generalizability of the findings to other age groups or socio-economic statuses. Additionally, the self-reported nature of the data could introduce biases like social desirability and recall bias. The study also did not account for the actual time spent watching or interacting with influencer content, which could provide more nuanced insights into the relationship between exposure and health outcomes. Lastly, this study focused solely on YouTube influencers, not considering influencers from other social media platforms, which might offer a different set of implications.

## Conclusions

In conclusion, this study offers valuable insights into the role of YouTube influencers in shaping health attitudes and behaviors among a Saudi Arabian audience. The data suggests that influencers do have a significant impact, particularly in the areas of diet and exercise. However, the public holds mixed opinions on whether these influencers always offer advice that is genuinely in the best interest of their audience. With healthcare influencers being the most popular, there is a clear opportunity for experts in the field to use the platform responsibly to improve public health. Given the limitations of the study, further research is warranted to explore these dynamics in a more nuanced and comprehensive manner.

## References

[1] Khan ML (2017). Social media engagement: what motivates user participation and consumption on YouTube?. Comput Hum Behav.

[2] Balakrishnan J, Griffiths MD (2017). Social media addiction: what is the role of content in YouTube?. J Behav Addict.

[3] (2023). Essential YouTube Statistics and Trends for 2023. https://datareportal.com/essential-youtube-stats.

[4] (2024). Number of YouTube Users Worldwide from 2020 to 2029. https://www.statista.com/forecasts/1144088/youtube-users-in-the-world.

[5] (2023). Digital in Saudi Arabia. Digital.

[6] (2024). Saudi Arabia (KSA) Social Media Statistics. Statistics.

[7] Osman W, Mohamed F, Elhassan M, Shoufan A (2022). Is YouTube a reliable source of health-related information? A systematic review. BMC Med Educ.

[8] Sokolova K, Perez C (2021). You follow fitness influencers on YouTube. But do you actually exercise? How parasocial relationships, and watching fitness influencers, relate to intentions to exercise. J Retail Consum Serv.

[9] Castonguay J, Messina N (2022). YouTube influencers: a new defense against childhood obesity?. Journ of Food Prod Mrktg.

[10] Harris J, Atkinson A, Mink M, Porcellato L (2021). Young people’s experiences and perceptions of YouTuber-produced health content: implications for health promotion. Health Educ Behav.

[11] Balcombe L, Leo DD (2023). The impact of YouTube on loneliness and mental health. Inform 2023.

[12] Chen J, Wang Y (2021). Social media use for health purposes: systematic review. J Med Internet Res.

[13] (2023). Raosoft Sample Size Calculator. http://www.raosoft.com/samplesize.html.

[14] Rowley J, Johnson F, Sbaffi L (2017). Gender as an influencer of online health information-seeking and evaluation behavior. J Assoc Inf Sci Technol.

[15] IBM Corp. (2024). How to Cite IBM Spss Statistics or Earlier Versions of SPSS. Computer software, IBM Corp..

[16] Llorente A, Garcia-Herranz M, Cebrian M, Moro E (2015). Social media fingerprints of unemployment. PLoS One.

[17] De Jans S, Hudders L, Naderer B, De Pauw V (2022). Impact of thin-ideals in influencer posts promoting healthy vs. unhealthy foods on tweens’ healthy food choice behavior. Front Psychol.

[18] Edwards ES, Sackett SC (2016). Psychosocial variables related to why women are less active than men and related health implications. Clin Med Insights Womens Health.

[19] Li W, Ding H, Xu G, Yang J (2023). The impact of fitness influencers on a social media platform on exercise intention during the COVID-19 pandemic: the role of parasocial relationships. Int J Environ Res Public Health.

[20] Tricás-Vidal HJ, Vidal-Peracho MC, Lucha-López MO, Hidalgo-García C, Monti-Ballano S, Márquez-Gonzalvo S, Tricás-Moreno JM (2022). Impact of fitness influencers on the level of physical activity performed by Instagram users in the United States of America: analytical cross-sectional study. Int J Environ Res Public Health.

[21] Durau J, Diehl S, Terlutter R (2022). Motivate me to exercise with you: the effects of social media fitness influencers on users’ intentions to engage in physical activity and the role of user gender. Digit Health.

[22] Alfaya MA, Abdullah NS, Alshahrani NZ (2023). Prevalence and determinants of social media addiction among medical students in a selected university in Saudi Arabia: a cross-sectional study. Healthcare (Basel).

